# Copper Micro-Flowers for Electrocatalytic Sensing of Nitrate Ions in Water

**DOI:** 10.3390/s24144501

**Published:** 2024-07-11

**Authors:** Roberta Farina, Giuseppe D’Arrigo, Alessandra Alberti, Silvia Scalese, Giuseppe E. Capuano, Domenico Corso, Giuseppe A. Screpis, Maria Anna Coniglio, Guglielmo G. Condorelli, Sebania Libertino

**Affiliations:** 1 IMM–CNR, Strada VIII Z.I., 5, 95121 Catania, Italy; giuseppe.darrigo@imm.cnr.it (G.D.); alessandra.alberti@imm.cnr.it (A.A.); silvia.scalese@imm.cnr.it (S.S.); giuseppeemanuele.capuano@imm.cnr.it (G.E.C.); domenico.corso@imm.cnr.it (D.C.); 2 Dipartimento di Scienze Chimiche, Università Degli Studi di Catania, Viale A. Doria 6, 95125 Catania, Italy; guido.condorelli@unict.it; 3 Dipartimento di Scienze Mediche, Chirurgiche e Tecnologie Avanzate “G.F. Ingrassia”, Università Degli Studi di Catania, Via S. Sofia 87, 95123 Catania, Italy; giuseppe.screpis@studium.unict.it (G.A.S.); ma.coniglio@unict.it (M.A.C.)

**Keywords:** nitrate detection, electrochemical sensor, screen-printed electrodes, electrocatalytic reaction, electrodeposition

## Abstract

The progressive increase in nitrate’s (NO_3_^−^) presence in surface and groundwater enhances environmental and human health risks. The aim of this work is the fabrication and characterization of sensitive, real-time, low-cost, and portable amperometric sensors for low NO_3_^−^ concentration detection in water. Copper (Cu) micro-flowers were electrodeposited on top of carbon screen-printed electrodes (SPCEs) via cyclic voltammetry (with voltage ranging from −1.0 V to 0.0 V at a scan rate of 0.1 V s^−1^). The obtained sensors exhibited a high catalytic activity toward the electro-reduction in NO_3_^−^, with a sensitivity of 44.71 μA/mM. They had a limit of detection of 0.87 µM and a good dynamic linear concentration range from 0.05 to 3 mM. The results were compared to spectrophotometric analysis. In addition, the devices exhibited good stability and a maximum standard deviation (RSD) of 5% after ten measurements; reproducibility, with a maximum RSD of 4%; and repeatability after 10 measurements with the RSD at only 5.63%.

## 1. Introduction

Nitrogen (N) is an essential macro element for the development of life on earth, as it is present in protein substances, chlorophyll, nucleic acids, etc., and participates in the constitution of most organisms’ tissues. Plants absorb nitrogen from the soil mainly in inorganic forms: nitric and ammoniacal. The ammonium ion binds to the cation exchange complex of the soil; therefore, the ammonium ion is retained and acts more slowly, influenced by microbial activity. The nitrate ion is immediately assimilable, as it is not retained by the soil colloids. This mobility makes it more available for absorption but also more prone to leaching and percolation in the presence of water surpluses. The leached nitrate nitrogen quickly reaches the deeper layers of the soil, becoming unreachable by plant roots and polluting the groundwater and rivers [1]. This causes risks to the environment and human health. The excessive presence of nitrates in drinking water is harmful to our body since they transform into nitrites that bind to hemoglobin to form methemoglobin, which then hinders oxygen transport and flux to tissues in the body [2]. On the other hand, the excess of nitrogen compounds in wastewater causes, with phosphorus, eutrophication, i.e., the uncontrolled proliferation of autotrophic species, especially algae, which become waste for aquatic environments. For these reasons, the World Health Organization (WHO) and European directives set the maximum contaminant level of NO_3_^−^ in public drinking water to 50 mg/L (~0.8 mM) [3]. To date, several methodologies have been developed to monitor nitrate concentration in water, such as flow injection analysis, spectrophotometry, chemiluminescence, capillary electrophoresis, ion chromatography, high-performance liquid chromatography, and gas chromatography-mass spectrometry [4]. Although these techniques are specific and sensitive, their use presents several drawbacks, such as the need for sampling and the use of sophisticated, expensive, and time-consuming tools. Therefore, substantial research has been oriented toward the development of alternative detection methods that are inexpensive and provide a quick response while still maintaining good sensitivity and selectivity. Electrochemical sensors with screen-printed electrodes (SPEs) are the best candidates. Using screen-printing technology, electrodes with reproducible chemical performances can be developed. Portable systems can be fabricated owing to the small size of SPEs, their linear output, low-power demand, rapid response, high sensitivity, and operating capacity at room temperature [5,6,7]. A screen-printed electrochemical cell is composed of three electrodes: the working electrode (WE), the reference electrode (RE), and the counter electrode (CE), printed on a low-cost solid substrate, often made of ceramic or plastic. The inks used for printing determine the properties of the electrochemical cell, and the appropriate modification of the working electrode surface plays a key role in the development of sensitive and selective electrochemical sensors for molecules/target substance detection [8]. The miniaturized design allows these electrochemical cells to be portable and suitable for on-site measurements and real-time analysis, avoiding the use of large amounts of reagents and samples. All these characteristics agree with green analytical chemistry principles [9,10]. To date, various electrochemical sensors (potentiometric, amperometric, and conductometric [11,12,13,14,15]) have been used to detect NO_3_^−^. Among copper (Cu), platinum (Pt), silver (Ag), and gold (Au), Cu was proven to be one of the most effective metals for catalyzing the electroreduction in NO_3_^−^ [16,17]. This is mainly due to its high conductivity (5.8 × 10^7^ S/m), which improves charge transfer [18]. In addition, Cu is the most cost-effective [19] compared to other electrocatalytic metals. Recently, researchers showed that increasing the working electrode electroactive surface area with copper deposition improves the limit of detection (LoD) of electrochemical NO_3_^−^ sensors [20,21,22]. For instance, a flexible screen-printed amperomeric electrochemical NO_3_^−^ sensor was developed and functionalized with Cu metal nanoclusters electrodeposited on Ag working electrodes. It showed a high capability to detect NO_3_^−^ in water with a low calculated LoD 0.207 nM (or 0.012 μg/L) and a dynamic concentration range from 50 to 500 μM (or 31 to 310 mg/L) using linear sweep voltammetry (LSV) [23]. In addition, nitrate detection was investigated using commercial single-walled carbon nanotube-modified Cu and Pd-Cu electrodes in a sulfuric acid solution by LSV. An extended concentration range (0.1 to 7.8 mM) and an LoD of 52 µM were obtained [24]. Therefore, starting from that research, we electrochemically deposited Cu micro-flowers on top of a commercially available carbon electrode using cyclic voltammetry (CV), thus obtaining a low-cost sensor. CV is a powerful electrochemical technique used to study chemical reactions initiated by electron transfer, which includes catalysis [25,26]. Micro-flowers were electrochemically characterized, and their structures were examined using scanning electron microscopy (SEM) and X-ray diffraction (XRD). The sensor showed an LoD of 0.87 µM for NO_3_^−^ in water and a wide dynamic concentration range, from 0.05 to 3.00 mM (3.1 to 186 mg/L), using LSV. Additionally, sensor stability over time, reproducibility, and repeatability were investigated. Finally, measurements obtained using the developed sensor were compared with those achieved using UV-visible spectrophotometry.

## 2. Materials and Methods

### 2.1. Chemicals and Apparatus

Chemicals in this work (all of them are of an analytical grade) were used without further purification. Sodium nitrate (NaNO_3_), copper sulfate pentahydrate (CuSO_4_·5H_2_O), and potassium chloride (KCl) were purchased from Merck KGaA (Darmstadt, Germany). milliQ water (resistivity of at least 18.2 MΩ cm) obtained by Simplicity UV (Millipore, by Merk, Darmstadt, Germany) was used for the preparation of all solutions. Screen-printed carbon electrodes (SPCE, cod. Ref. 150) were bought from Metrohm DropSens s.r.l. (Origgio, VA, Italy). Copper electrodeposition and all electrochemical measurements were performed by the Palmsens4 electrochemical workstation by PalmSens BV (C-PS4-BP.F2.10, GA Houten, The Netherlands). An UV-vis spectrophotometer (Varian Cary 50, Palo Alto, CA, USA) was used to compare the electrochemical data with a standard measurement. Scanning electron microscopy (SEM) was conducted using an e-beam lithography apparatus Raith 150 (Dortmund, Germany) in the SEM operation mode. EDX spectra were obtained by an energy-dispersive X-ray microanalysis system (X-MAX, 80 mm^2^ by Oxford Instruments, Abingdon, UK) inside a ZEISS FE-SEM SUPRA 35 (Carl Zeiss AG, Jena, Germany). X-ray diffraction patterns were collected using Smartlab equipment made by Rigaku (Sevenoaks, UK).

Electrochemical measurements were performed in milliQ water solutions containing KCl 0.1 M. Spectrophotometric measurements were performed by measuring the nitrate peak height at 205 nm in the same electrochemical solutions.

### 2.2. Copper Micro-Flowers Electrodeposition and Storage

Copper was CV electrochemically deposited on an SPCE (4 mm diameter) surface by performing 5 cycles in the potential range from 1.0 to 0.0 V at a scan rate of 0.1 V s^−1^, and 0.1 M CuSO_4_ in 0.1 M KCl, supporting the electrolyte solution [27]. The electrochemical cell of SPCE is a three-electrode composed of single-sided carbon WE, silver RE, and platinum CE. The solution was not stirred during deposition. Each cycle lasted roughly 2 min. This electrochemical process created Cu micro-flowers on the carbon surface [28]. Once fabricated, the electrodes were either used immediately or stored under a nitrogen atmosphere to avoid Cu oxidation. After each measurement, the electrodes were rinsed in milliQ water, dried under N_2_ flux, and stored in an N_2_ atmosphere.

### 2.3. Electrochemical Characterization

**Figure 1:**
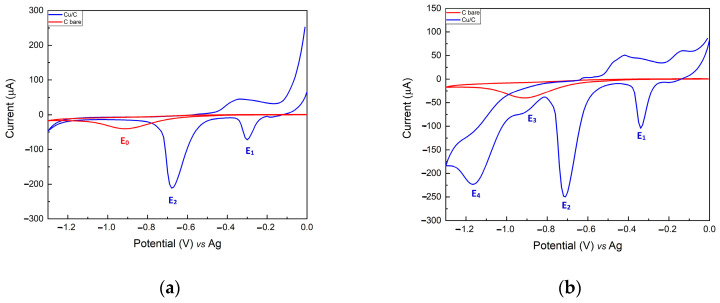
(**a**) Cyclic voltammograms in 0.1 M KCl supporting the electrolyte of (**a**) the bare C electrode (red trace) and electrodeposited Cu/C electrode (blue trace); (**b**) the bare C electrode (red trace) and an electrodeposited Cu/C electrode (blue trace) in the presence of 1.6 mM NO_3_^−^.

Figure 1a shows the CV of a bare carbon electrode (red trace) and modified Cu/C electrode (blue trace) performed in a 0.1 M KCl electrolyte solution. E_0_ is the characteristic peak of the SPCE, which flattens in the case of the modified electrode, proving the coverage. Compared to the bare C electrode, the modified electrode shows two cathodic peaks at −0.33 V (E_1_) and −0.75 V (E_2_), characteristic of Cu(I) and Cu(II), respectively. Their presence confirms the success of the copper electrodeposition process.

Figure 1b shows the CV of a bare carbon electrode (red trace) and of a modified Cu/C electrode (blue trace) performed in the presence of nitrate (1.6 mM NO_3_^−^) in an electrolyte solution (0.1 M KCl). The bare C electrode CV remained unchanged, while two additional reduction peaks at −0.86 V (E_3_) and −1.08 V (E_4_) appeared in the Cu/C electrode CV. These peaks can be ascribed to the reduction in NO_3_^−^ (peak E_3_) and NO_2_^−^ (peak E_4_) ions.

Calibration curve measurements and reproducibility tests were performed on solutions containing NO_3_^−^ to 0.05, 0.15, 0.5, 0.8, 1.5, 1.8, and 3.0 mM. The same solutions were used for electrochemical and spectrophotometric measurements. After each measurement, lasting roughly 10 s, the electrode was rinsed in milliQ water and dipped in the solution for the next measurement. Time stability was measured using a 2 mM NO_3_^−^ solution and performing ten measurements in a three-day timeframe.

## 3. Results

### 3.1. Morphological Characterization

**Figure 2:**
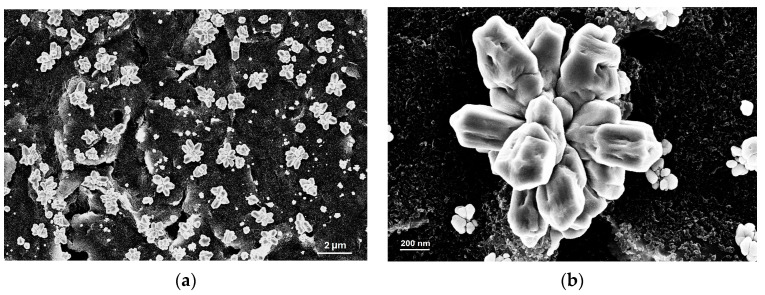
SEM images of screen-printed C WE modified with 5 electro-deposition cycles of Cu. (**a**) Large area image (marker 2 µm); (**b**) magnification of a single Cu flower (marker 200 nm).

Scanning electron micrographs were acquired to study the C surface and the electrodeposited copper morphologies. Figure 2a shows the uniform distribution of Cu micro grains all over the SPCE electrode surface. The carbon surface is still visible, suggesting that electrode porosity is preserved. Figure 2b shows the magnification of the Cu grain structures obtained after five Cu electrodeposition cycles on the WE. Flower-shaped crystals with a specific orientation were deposited. Cu micro-flowers have a high surface-to-volume ratio from 0.01 to 1 along a well-defined orientation (see after) with a profile width ranging from 0.5 to 4.5 μm (Figure S1).

The flower-like structure of the crystallites favors a greater catalytic effect. Their formation is associated with the anion-induced directional growth of Cu crystal planes [29]; this effect fully exposes the active sites and increases the synergetic catalytic efficiency [30,31]. To evaluate and quantify the elemental composition of the bare carbon WE and the modified Cu/C WE, energy-dispersive X-ray spectroscopy (EDS) analysis was performed. The estimated composition of the WE surface revealed % weight values of 90.40% of C, 2.54% of O, 0.36% of Si, 6.86% of Cl (Figure S2) for the bare carbon WE, and % weight values of 70.05% Cu, 21.10% Cl, 6.98% C, and 1.88% O (Figure S3) for the modified Cu/C electrode. Notably, the percentage values obtained for each element provided an average surface value of the sample since the Cu structures did not form a fully homogeneous and flat coverage on the bare carbon WE. Hence, the obtained values were in the expected range and revealed that most of the electrode surface is covered with electrodeposited Cu.

**Figure 3:**
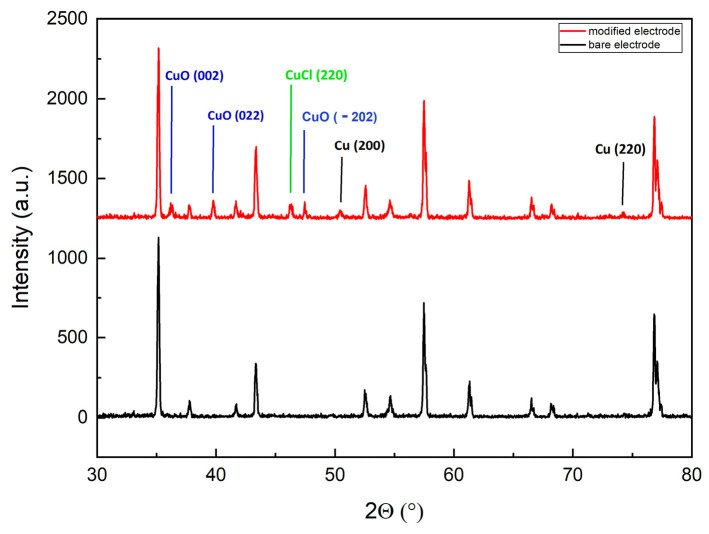
XRD pattern of bare and modified electrodes with 5 CV cycles of copper deposition.

Cu deposited on the surface of the WE was characterized by X-ray diffraction (XRD) to evaluate its crystallographic structure. The XRD pattern of the bare electrode and modified electrode with five CV cycles of copper deposition are shown in Figure 3.

The modified electrode shows peaks at positions (2θ) of 50.48° and 74.13° corresponding to the Bragg reflections of crystalline Cu(200) and Cu(220), and peaks at positions (2θ) of 36.18°, 39.76°, 47.46° corresponding to CuO(002), CuO(022), and CuO(−202), respectively. The modified electrode shows peaks at positions (2θ) of 46.3°, corresponding to CuCl(220). This pattern confirms the successful coverage with crystalline Cu on top of the C WE. Additionally, the observation is analogous to that of Inam et al. [23] and Chen et al. [32], who demonstrated that the morphology of Cu electro-crystallization is driven by the surface energy differences in the crystallographic planes. The high-index facet exhibits a high density of low-coordinated atoms, providing more catalytic sites for electrocatalysts [33].

Various Cu crystallographic orientations exhibit distinct surface energies, impacting thermodynamic stability, nitrate molecule adsorption capacity, and reactivity. Additionally, different crystallographic orientations present varying densities of active sites, affecting the number of sites available for catalytic reactions. Metallic copper is known to enhance nitrate reduction due to its high electrical conductivity and abundant conduction electrons. In contrast, the presence of chlorine can adversely affect the nature of the active sites on the surface and their interactions with nitrates.

Therefore, XRD measurements provide information, besides Cu, on all the compounds present in the sample. Their formation is associated with the anion-induced directional growth of copper crystal planes; this effect fully exposes the active sites and increases the synergetic catalytic efficiency. The atomic arrangement of different crystal planes can influence the adsorption of reaction intermediates, thereby impacting the activity and selectivity of electrocatalysts for NO_3_^−^ [33,34,35,36,37,38].

### 3.2. Study of the Electrochemical Reaction on Electrodes

The nature of the electrochemical reaction taking place on the Cu/C electrode was studied. The energy levels of their highly occupied d-orbitals were closely aligned with the lowest unoccupied molecular π* orbital of NO_3_⁻, facilitating efficient electron transfer at the catalyst surface. Therefore, the effect of the scanning speed, from 50 to 500 mVs^−1^, on the NO_3_^−^ reduction peak current was examined [33].

**Figure 4:**
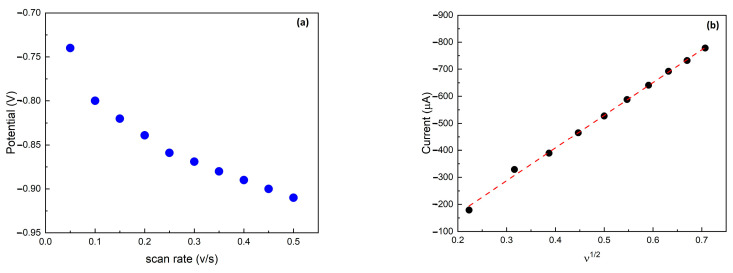
(**a**) The NO_3_^−^ reduction peak potential as a function of the scan rate. (**b**) Maximum peak current as a function of the square root of the scan rate. The dashed red line is the linear best fit of the data.

Figure 4a shows that the potential (E_p_) at which the NO_3_^−^ reduction occurs shifts negatively when the scan rate increases. This characteristic behavior is associated with a diffusion-controlled irreversible electron transfer process [31]. Figure 4b summarizes the cathodic peak current as a function of the square root of the scan rate. It shows the cathodic peak current increased linearly with the scan rate, indicating a diffusion-controlled reduction process, as described by the Randles–Ševčík equation [39] (Equation (1)):

(1) $$i_{p}=-2.69\times 10^{5}\;n^{\frac{3}{2}}\;AD_{0}^{\frac{1}{2}}\;v^{\frac{1}{2}}\;C$$

where *i_p_* is the peak current, *n* is the number of electron transfers (2 for NO_3_^−^), *A* is the active surface area (cm^2^), *D*_o_ is the diffusion coefficient (2.0 × 10^−6^ cm^2^ s^−1^ for NO_3_^−^), *v* is the scan rate (V s^−1^), and C is the NO_3_^−^ concentration (mol cm^−3^). The Randles–Sevcik equation is a tool in electrochemistry that allows us to relate three key parameters: surface area, diffusion coefficient, and redox concentration. By employing this equation, the effective electrochemical surface areas of both C and Cu/C WE were calculated, obtaining values of 0.0197 cm^2^ and 0.0404 cm^2^, respectively, showing that the Cu electrodeposition induces an increase in the effective surface of more than 100%. To study the kinetics of the reaction on an electrode, the half-peak potential (E_p/2_), defined as the potential corresponding to *i_p_*_/2_, is usually examined [40]. Figure S5 shows the ΔE_p/2_ = E_p_ − E_p/2_ calculated and plotted as a function of the scan rate. These data indicate that the transfer coefficient of the reduction reaction is independent of the scan rate.

### 3.3. Electrochemical Sensor Performance for Nitrate Ion Detection

To study the electrochemical sensor analytical performances, linear sweep voltammetry was used to detect NO_3_^−^ in a 0.1 M KCl electrolyte solution (pH = 7). The KCl electrolyte solution is an excellent medium since it allows the NO_3_^−^ content to be directly measured in water without changing the electrolytes’ pH or interfering with the sensor’s performance. The average of three measurements of the NO_3_^−^ reduction peak current at 0.86 V over the 0.05–3.0 mM concentration range was used to construct a calibration plot. The data show a linear detection range from 0.05 to 3 mM with a sensitivity of 44.71 μA/mM and a coefficient of determination (R^2^) of 99.28% (N = 3) (Figure S6). Based on a signal-to-noise ratio of three, the limit of detection (LoD) was estimated to be 0.87 µM. It can be seen that the Cu(II) reduction peak amplitude increased linearly with the nitrate concentration. This observation can be explained by the catalytic effect of the copper. Filimonov et al. [41] demonstrated that cuprous ions exhibit a catalytic effect with nitrate since they are electrochemically active and, as a result, an increase in the mass transport and the electron transfer process can be obtained.

**Figure 5:**
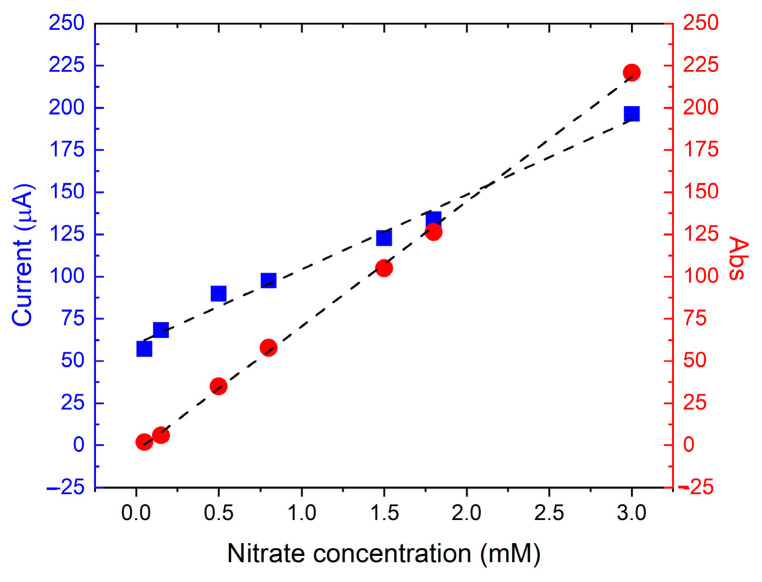
Calibration curve for nitrate detection for UV-vis spectrophotometer (red circles refer to the right axis) and the Cu/C sensor (blue squares refer to the left vertical axis). Each point represents the average peak current performed by three measurements.

Measurements performed using the reported sensor were compared with absorbance measurements by UV-vis spectrophotometry. The measurements obtained by the two instruments are summarized in Figure 5. The calibration plot for red circles refers to the right axis as the spectrophotometer dataset, and the blue squares refer to the left vertical axis and represent the average of three peak currents. The data show that the electrochemical sensor has a sensitivity only 1.61 times lower than that of the spectrophotometer one, demonstrating the good quality of the developed sensor [42].

### 3.4. Reproducibility, Repeatability, and Stability of the Sensor

**Figure 6:**
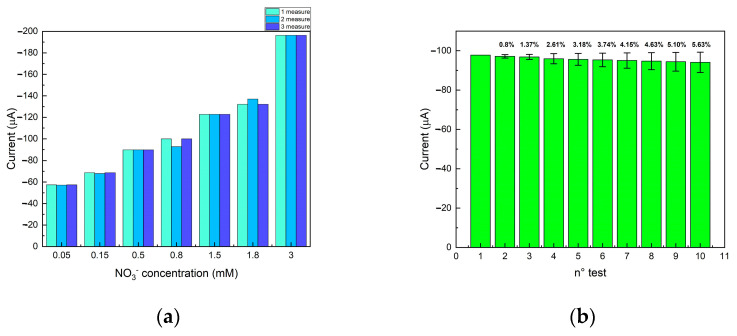
(**a**) Reproducibility test for Cu/C sensor. NO_3_^−^ peak current as a function of NO_3_^−^ concentration (mM); (**b**) Repeatability test for Cu/C sensor. The reduction peak current of the NO_3_^−^ was acquired from the same sample by repeating the measurement 10 times. The percentage reported in the Figure is the difference between the successive measurements from the first one.

Reproducibility, repeatability, and sensor stability were evaluated. Reproducibility was studied by carrying out measurements on the same solution with three different electrodes manufactured using the same procedure; instead, repeatability was studied by carrying out repeated measurements with the same electrode on the same solution. Electrodes were rinsed with milliQ water and dried with compressed air before performing each test. For the reproducibility test, the electrodes were tested at seven different concentrations of NO_3_^−^ (0.05, 0.15, 0.5, 0.8, 1.5, 1.8, and 3.0 mM); the measurements at each concentration were carried out three times. The relative standard deviations (RSDs) were 0.15%, 0.39%, 0%, 4.89%, 0.02%, 2.83%, and 0%, respectively, demonstrating good reproducibility, as shown in Figure 6a (The table containing all the data is reported in the Supplementary Material, Table S1). This result was achieved owing to the good quality of the functionalization process, which ensures excellent homogeneity and reproducibility of the deposition of the sensitive material.

The repeatability test was carried out using the same electrode (10 consecutive measurements) in the same NO_3_^−^ (0.8 mM) solution. The RSD was calculated by comparing the amplitude of the reduction peak current of the subsequent measurements with the first one. The concentration of 0.8 mM was selected as a reference since it was set as the maximum limit accepted by the WHO and European directives. The calculated RSDs were 0%, 0.8%, 1.37%, 2.61%, 3.18%, 3.74%, 4.15%, 4.63%, 5.10%, and 5.63%, respectively, for the seven NO_3_^−^ concentrations, demonstrating good repeatability, as shown in Figure 6b. In addition, the sensor had a stable behavior up to the tenth measurement, characterized by a reduction of 5% in the amplitude of the NO_3_^−^ cathodic peak compared to the first measurement. This ensured that the sensor could be reused but only for a limited number of measurements. This result is promising because the sensor developed in this work shows greater reproducibility than the works reported in the literature to our knowledge [23]. Stability over time was measured using a 2 mM NO_3_^−^ solution, indicating that the sensor, properly stored, is stable after three days of measurements.

Finally, the sensor can also be used in impure water. We tested the device using tap water as the solution medium. The data (shown in Figure S7 of the Supplementary Material) demonstrate that the medium purity does not affect the calibration curve, thus implying that none of the impurities contained in tap water affect the device’s performance.

## 4. Conclusions

In this work, a carbon screen-printed electrochemical sensor for NO_3_^−^ detection in water was fabricated by the electrodeposition of Cu and characterized both in terms of fabrication parameters and performances in detection. The device, obtained after five electrodeposition cycles, CV, partially covered the C surface with Cu micro-flowers, the presence of which increased the electrode active surface area by more than 100% compared to the bare carbon electrode. NO_3_^−^ has been quantitatively determined in the range extending from 0.05 to 3.00 mM with an estimated limit of detection of 0.87 µM in neutral media composed of a 0.1 M electrolyte solution of KCl. The sensor showed high reproducibility with a maximum RSD of 4%. The repeatability test confirmed that the same sensor shows an RSD of 5.63% after the tenth measurement on the same solution. In addition, the sensor is stable up to the tenth measurement, which is characterized by a reduction of 5% in the amplitude NO_3_^−^ cathodic peak compared to the first one. Measurements made with the proposed portable sensor were compared with absorbance measurements by UV-vis spectrophotometry, showing a sensitivity of only a factor 1.6 lower than the benchtop instrument. The proposed sensor also works with tap water; hence, it can be easily implemented in precision agriculture as a low-cost, fast, specific, and sensitive sensor for the detection of nitrate ions in irrigation water.

## 5. Patents

R. Farina and S. Libertino, *Nitrates electrocatalytic detection in water by copper micro-flowers*, under the patenting procedure, filed the 11 March 2024, n. 102024000005344, at the Italian “Ministero delle Imprese e del Made in Italy”.

## Data Availability

The raw data supporting the conclusions of this article are included in the paper and Supplementary Material figures and tables.

## Supplementary Materials

The following supporting information can be downloaded at https://www.mdpi.com/article/10.3390/s24144501/s1. Figure S1: Scanning electron micrographs of magnification images of screen-printed C WE modified through 5 cycles of Cu electrodeposition. Profile width estimation; Figure S2: EDS of screen-printed bare C WE; Figure S3: EDS of screen-printed C WE modified through 5 cycles of Cu electrodeposition; Figure S4: Reproducibility test for Cu/C sensor. NO_3_^−^ peak current as a function of NO_3_^−^ concentration (mM); Figure S5: ΔE_p/2_ as a function of the scan rate for nitrate reduction; Figure S6: Reproducibility test for Cu/C sensor. NO_3_^−^ peak current as a function of NO_3_^−^ concentration (mM); Figure S7: Calibration curves for NO_3_^−^ concentration added to milliQ water (blue dots) and to tap water (green squares) and Table S1: Measurements of peak current and relative standard deviation as a function of the nitrate concentration.
