# Supplementary material for: Copper Micro-Flowers for Electrocatalytic Sensing of Nitrate Ions in Water
Source: Sensors (Basel). 2024 Jul 11;24(14):4501. doi: 10.3390/s24144501 (PMC11280502; doi:10.3390/s24144501)
Supplement: Supplementary file 1 [file sensors-24-04501-s001.zip › sensors-3061871-supplementary.pdf]

SUPPLEMENTARY MATERIAL

Copper Micro-Flowers for Electrocatalytic Sensing of Nitrate Ions in Water

Roberta Farina [1,2,*], Giuseppe D'Arrigo [1], Alessandra Alberti [1], Silvia Scalese [1], Giuseppe E. Capuano [1], Domenico Corso [1], Giuseppe A. Screpis [3], Maria Anna Coniglio [1,3], Guglielmo G. Condorelli [2] and Sebania Libertino [1,*]

[1] CNR–IMM, Strada VIII Z.I., 5, 95121 Catania, Italy; giuseppe.darrigo@imm.cnr.it (G.D.); alessandra.alberti@imm.cnr.it (A.A.); silvia.scalese@imm.cnr.it (S.S.); giuseppeemanuele.capuano@imm.cnr.it (G.E.C.); domenico.corso@imm.cnr.it (D.C.); ma.coniglio@unict.it (M.A.C.)
[2] Dipartimento di Scienze Chimiche, Università Degli Studi di Catania, Viale A. Doria 6, 95125 Catania, Italy; guido.condorelli@unict.it
[3] Dipartimento di Scienze Mediche, Chirurgiche e Tecnologie Avanzate "G.F. Ingrassia", Università Degli Studi di Catania, Via S. Sofia 87, 95123 Catania, Italy; giuseppe.screpis@studium.unict.it
* Correspondence: roberta.farina@imm.cnr.it (R.F.); sebania.libertino@imm.cnr.it (S.L.)

1. Morphological Characterization

Figure S1 shows a Cu flower-shaped crystal electrodeposited after 5 cycles of voltammetric scans. The white line provides the width estimate of the flowers. The profile width is an important parameter for characterizing microstructure dimensions and surface characteristics.

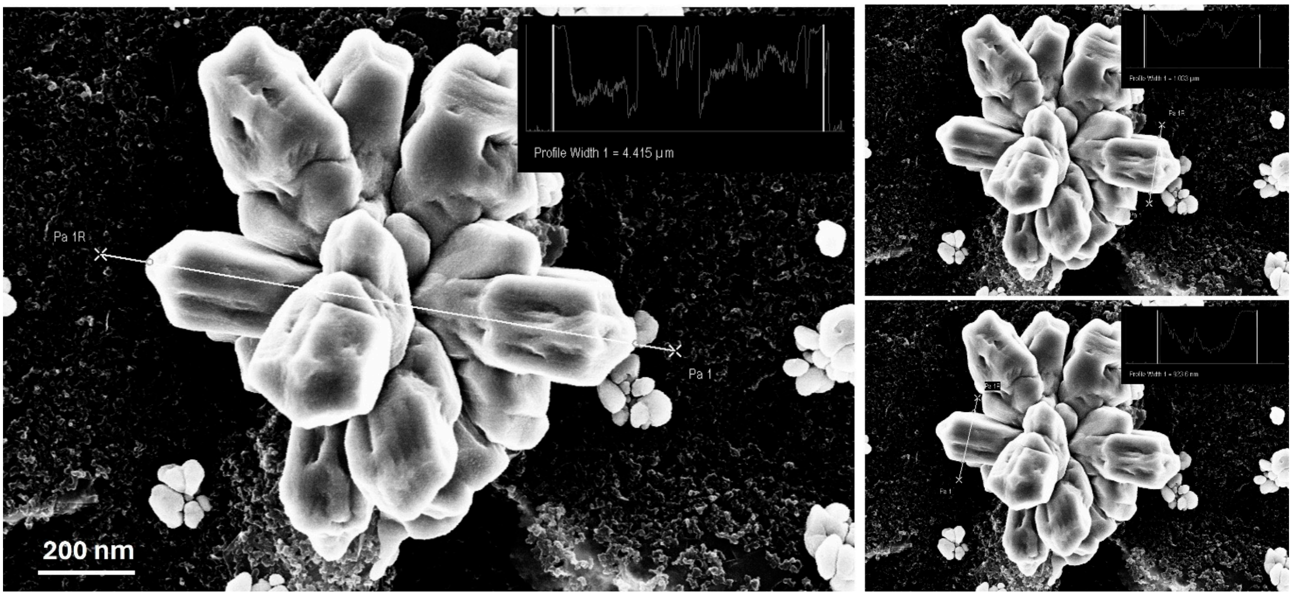

Figure S1. Scanning electron micrographs of magnification images of screen-printed C WE modified through 5 cycle of Cu electrodeposition. Profile width estimation.

Based on energy-dispersive X-ray spectroscopic analysis in Figure S2, the composition of the bare C WE surface was estimated to be 90.40% C, 2.54% O, 0.36% Si, and 6.86% Cl. Meanwhile, as shown in Figure S3, the composition of the screen-printed C WE after 5 cycles of Cu electrodeposition was estimated to be 70.05% Cu, 21.10% Cl, 6.98% C, and 1.88% O.

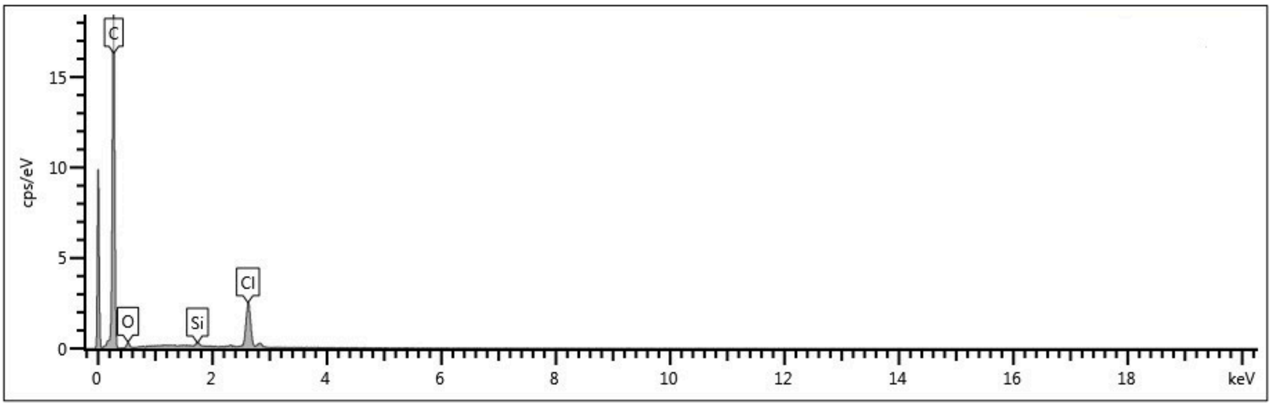

Figure S2. EDS of screen-printed bare C WE.

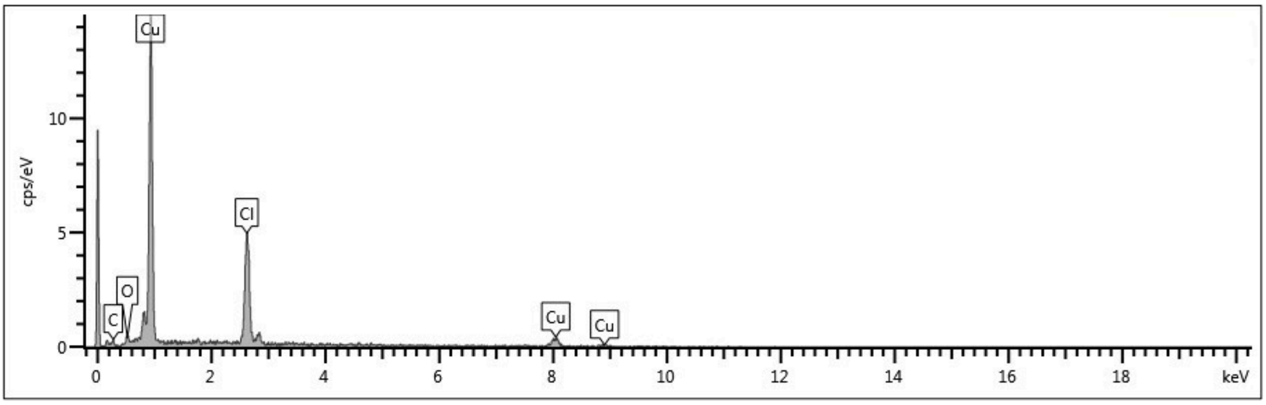

Figure S3. EDS of screen-printed C WE modified through 5 cycle of Cu electrodeposition.

2. Definition of the best electrodeposition cycle conditions

To define the best sensing electrode, the number of Cu electrodeposition cycles has been changed from 2 to 7. The sensor calibration curves for the three electrodes obtained after 2, 5, and 7 Cu electrodeposition cycles are shown in Fig. S4. The best device is obtained after 5 cycles since it exhibits the best sensitivity and has the highest current as a function of the NO_3^- concentration.

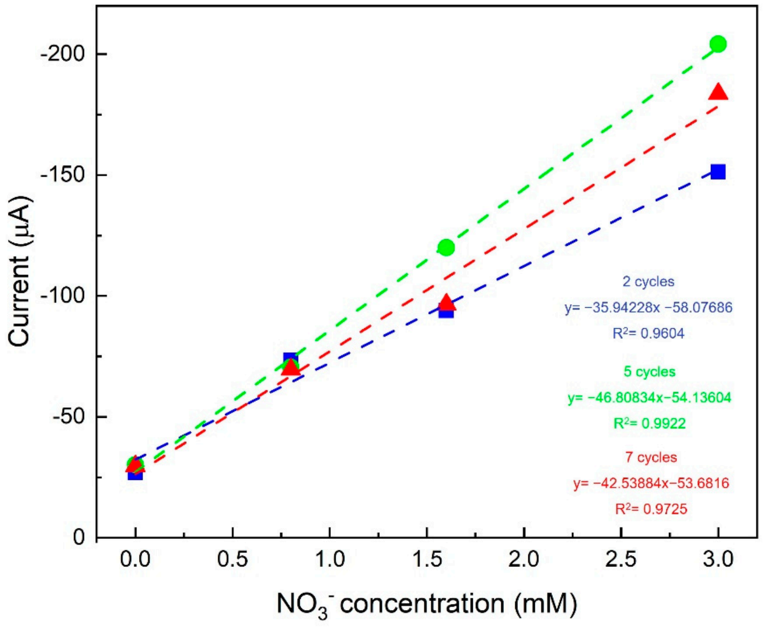

Figure S4. Calibration curves for sensors obtained after 2 cycles (blue squares), 5 cycles (green dots), and 7 cycles (red triangles) Cu electrodeposition.

3. Study of the kinetics reactions on electrodes

To study the kinetics of the reaction on an electrode, the half-peak potential ($E_{p/2}$), defined as the potential corresponding to $i_{p/2}$, is examined. E_p is the peak potential. Figure S5 shows the $\Delta E_{p/2} = E_p - E_{p/2}$ calculated and plotted as a function of the scan rate. The figure shows an average $\Delta E_{p/2}$ of 20 mV (standard deviation 2 mV, N = 3) over a scan rate range of 50 to 500 mV s^{-1}. This indicates that the transfer coefficient of the reduction reaction is independent of the scan rate.

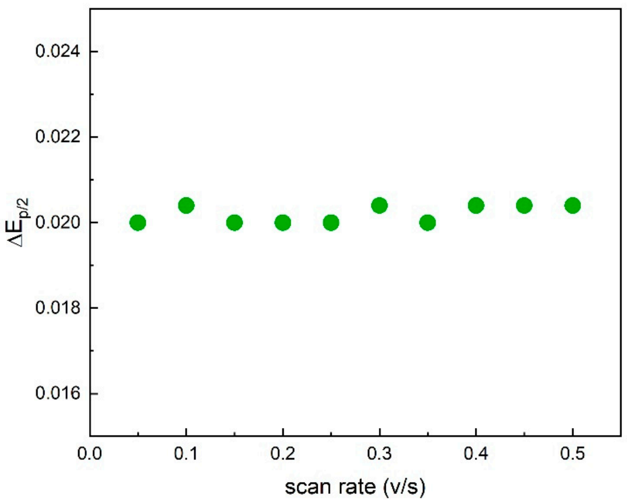

Figure S5. $\Delta E_{p/2}$ as a function of the scan rate for nitrate reduction.

4. Reproducibility, Repeatability, and Stability of the Sensor

To estimate reproducibility, the electrodes were tested at seven different concentrations of NO_3^- (see table T1): 0.05, 0.15, 0.5, 0.8, 1.5, 1.8, and 3.0 mM. The measurements at each concentration were carried out three times and the relative standard deviation (RSD) was measured. All the data are reported in Table T1 and summarized in Figure S6. A good reproducibility is obtained.

Table T1. Measurement of peak current and relative standard deviation as a function of the nitrate concentration.

Concentration (mM)	Measured Current (µA)			%		
	1	2	3			
0.05	-57,00	-57,00	-57,00	0.00	0.00	0.00
0.15	-68,64	-67,95	-68,64	0.57	0.39	0.41
0.5	-85,72	-85,72	-85,72	0.00	0.00	0.00
0.8	-98,93	-93,72	-98,92	4.04	4.16	4.47
1.5	-122,82	-122,77	-122,82	0.02	0.02	0.03
1.8	-132,16	-137,06	-132,18	2.11	2.83	2.8
3	-196,35	-196,35	-196,35	0.00	0.00	0.00

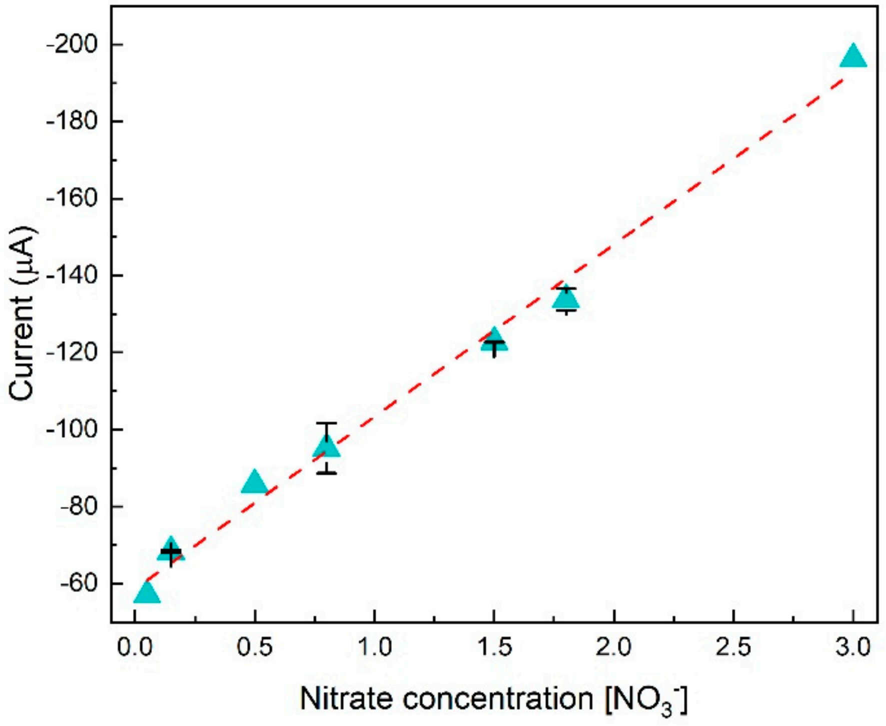

Figure S6. Reproducibility test for Cu/C sensor. NO_3^- peak current as a function of NO_3^- concentration (mM).

5. Interferences effect

To test if any of the possible interferences contained as impurities in tap water can affect the sensor performances we performed two calibration curves: the first using milliQ water with NO_3^- at various concentrations, and the second using tap water as solution medium. The data are summarized in Figure S7. The calibration data in milliQ water are shown as blue dots and the dashed red line is referred to those points, while the points obtained by adding tap water with NO_3^- at known concentrations are shown as green squares. The first tap water point has been obtained with two independent measurements: with our device and using spectrophotometric measurements. The same results, 0.1 mM, have been obtained with the two-system confirming that our device is not affected by the impurities contained in tap water.

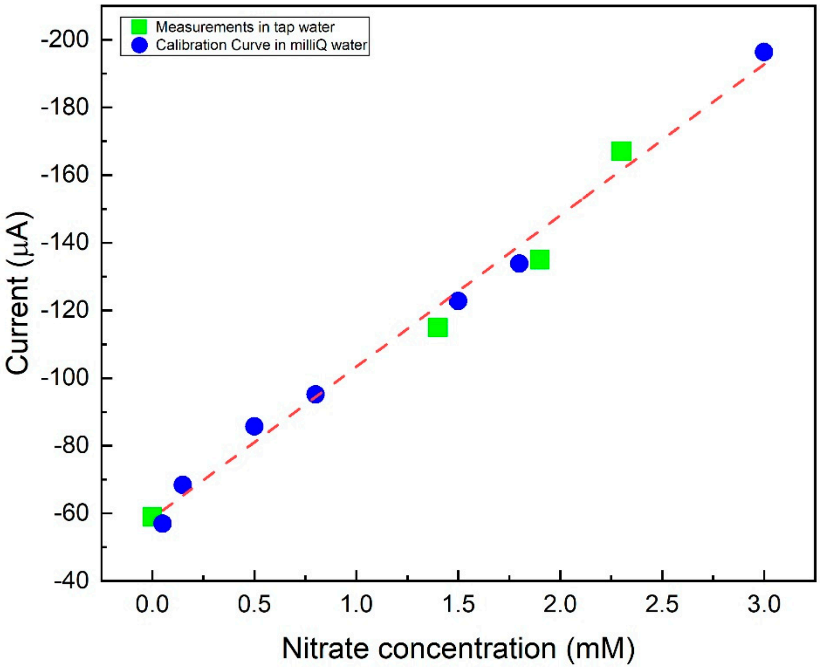

Figure S7. Calibration curves for NO_3^- concentration added to milliQ water (blue dots) and to tap water (green squares)
